# Alterations in Gut Glutamate Metabolism Associated with Changes in Gut Microbiota Composition in Children with Autism Spectrum Disorder

**DOI:** 10.1128/mSystems.00321-18

**Published:** 2019-01-29

**Authors:** Mingbang Wang, Jing Wan, Han Rong, Fusheng He, Hui Wang, Jiaxiu Zhou, Chunquan Cai, Yan Wang, Ruihuan Xu, Zhaoqing Yin, Wenhao Zhou

**Affiliations:** a Shanghai Key Laboratory of Birth Defects, Division of Neonatology, Children’s Hospital of Fudan University, National Center for Children’s Health, Shanghai, China; b Division of Neonatology, The People’s Hospital of Dehong Autonomous Prefecture, Mangshi, Yunnan, China; c Shenzhen Key Laboratory for Psychological Healthcare, Shenzhen Institute of Mental Health, Shenzhen Kangning Hospital, Shenzhen Mental Health Center, Shenzhen, China; d Imunobio, Shenzhen, Guangdong, China; e Xiamen branch of Children’s Hospital of Fudan University (Xiamen Children’s Hospital), Xiamen, Fujian, China; f Division of Psychology, Shenzhen Children’s Hospital, Shenzhen, Guangdong, China; g Division of Neurosurgery, Tianjin Children’s Hospital, Tianjin, China; h Clinical Laboratory, Longgang Central Hospital of Shenzhen, Guangdong, China; Vall d’Hebron Research Institute

**Keywords:** autism, glutamate, gut microbiota, liquid chromatography-mass spectrometry, metabolome, metagenome

## Abstract

Changes in the gut microenvironment may influence the pathogenesis of autism spectrum disorders (ASD). Here, we investigated the composition of the gut microbiota and metabolites in children with ASD. Ninety-two children with ASD and 42 age-matched children exhibiting typical development (TD) were enrolled in the two-stage study. In the discovery stage, shotgun metagenomic sequencing and liquid chromatography-mass spectrometry (LC-MS) were performed simultaneously on fecal samples obtained from 43 children in the ASD group and 31 children in the TD group. Systematic bioinformatic analyses were performed to identify gut metabolites associated with altered gut microbiota composition. At the validation stage, differential metabolites were tested using LC-MS with an additional 49 and 11 children in the ASD and TD groups, respectively. Altered glutamate metabolites were found in the ASD group, along with a decline in 2-keto-glutaramic acid and an abundance of microbiota associated with glutamate metabolism. These changes in glutamate metabolism were correlated with lower levels of the highly abundant bacteria Bacteroides vulgatus and higher levels of the potentially harmful Eggerthella lenta and Clostridium botulinum. Lower gut cortisol levels have also been identified in the ASD group and associated with changes in gut microbiota glutamate metabolism. Finally, gut 2-keto-glutaramic acid was validated as a potential biomarker for ASD. The significant changes in the gut microenvironment in children with ASD may provide new insight into the cause of ASD and aid in the search for diagnostic and therapeutic approaches.

**IMPORTANCE** Multiple lines of evidence suggest that the gut microbiota may play an important role in the pathogenesis of ASD, but the specific mechanism is still unclear. Through a comprehensive gut metagenomic and metabolome study of children with ASD, alterations in gut metabolite composition were found in children with ASD, and these alterations were linked to changes in gut microbiota composition. This may give us a deeper understanding of the role of gut microbiota in the pathogenesis of ASD.

## Abstract (precis)

Multiple lines of evidence suggest that the gut microbiota may play an important role in the pathogenesis of ASD, but the specific mechanism is still unclear. Through a comprehensive gut metagenomic and metabolome study of children with ASD, alterations in gut metabolite composition were found in children with ASD, and these alterations were linked to changes in gut microbiota composition. This may give us a deeper understanding of the role of gut microbiota in the pathogenesis of ASD.

## INTRODUCTION

Autism spectrum disorders (ASD) affect 2.24% of children aged 3 to 17 years in the United States (1), and there is currently no feasible laboratory diagnostic or effective cure for the condition (2). Multiple lines of evidence have shown that the gut microbiota may play an important role in the pathogenesis of ASD through the “gut-brain” axis (3); in patients with ASD, changes in the gut environment, initiated through a fecal microbiota transplantation (FMT), can improve the core symptoms of ASD (4). Indeed, in the clinical trial conducted by Kang et al., after an open-label fecal transplantation in 18 patients with ASD, sixteen of eighteen (88.8%) patients had a significant improvement in GI problems, and the core syndromes of ASD were also significantly improved (4). In the ASD mouse model, which exhibits abnormal gut metabolites, fecal transplantation can correct the metabolite abnormalities and improve behavioral abnormalities in these mice (5). An increasing number of studies have shown that the gut metabolites also play an important role in ASD (5). For example, the ASD mouse model showed a significant increase in the level of the metabolite 4-ethylphenol sulfate, which can be lowered by administration with Bacteroides fragilis (5), whereas other gut metabolites, such as short-chain fatty acids (SCFAs), which originate from microbiota, can regulate blood-brain barrier permeability and affect animal behavior (6). These studies suggest that changes in the gut microenvironment in patients with ASD may play an important role in the pathogenesis of the disorder. However, most of the current research focuses on understanding the taxonomic composition of the gut microbiota in these patients and rarely simultaneously explores the composition of gut metabolites (3). Therefore, studying the gut microbiota and metabolite composition simultaneously in patients with ASD could be critical to our understanding of the etiology of ASD.

In the present study, fecal samples were collected from children with ASD or those exhibiting typical development (TD), and shotgun metagenomic sequencing and liquid chromatography-mass spectrometry (LC-MS)-based metabolomics were performed to identify gut metabolites and microbiota composition associated with ASD.

## RESULTS

### Subject characteristics.

A total of 92 children with ASD and 42 children exhibiting TD were enrolled in the study (as detailed in Table S1 in the supplemental material). Demographic information and statistical analyses are shown in Table 1. A flowchart of our study design and analysis is shown in Fig. S1 in the supplemental material. In short, the study was divided into two stages. The gut metagenome and metabolome studies were carried out at the discovery stage with 42 children in the ASD group and 31 children in the TD group. At the validation stage, an additional 49 children with ASD and 11 children with TD were included for the metabolome study.

**TABLE 1:** Demographic and clinical characteristics of study subjects

Characteristic	Value for characteristic at the discovery stage		*P* value^*a*^	Value for characteristic at the validation stage		*P* value
	ASD	TD		ASD	TD	
No. of subjects	43	31	NA	49	11	NA
Gender (no. of females/no. of males)	7/36	14/17	0.0183274	7/42	6/5	0.008637198
Age (yr)	4.51 ± 2.23	3.14 ± 1.73	0.0515	5 ± 2.8	6.8 ± 3.7	0.149023209
Method(s)	Metagenome and LC-MS/MS	Metagenome and LC-MS/MS	NA	LC-MS/MS	LC-MS/MS	NA
GI problems (no. of subjects without GI problems/no. of subjects with GI problems)	24/19	0/31	NA	NA	NA	NA
IgA (OD_450_)	1.62 ± 0.24	1.24 ± 0.38	0.0000163	NA	NA	NA

a NA, not applicable.

10.1128/mSystems.00321-18.1 FIG S1 Flowchart of the study design. Shotgun metagenome sequencing and metabolome analyses were performed on fecal samples of 43 children with ASD and 31 children with TD to obtain the gut metabolite composition and microbiota composition at the taxonomic and functional levels. Significant differences in metabolites, pathways, and taxons were identified between the ASD and TD groups. Fecal samples of an additional 49 and 11 children in the ASD and TD groups were collected to determine whether the differential metabolite was a potential marker for children with ASD. Abbreviations: ASD, autism spectrum disorder; ESI, electrospray ionization; LC-MS/MS, liquid chromatography-tandem mass spectrometry; MassLynx 4.1, mass spectrometry software version by Waters company; MEGAN5, MEtaGenome Analyzer version 5; MWAS, metagenome-wide association study; PCA, principal-component analysis; TD, typical development; yr, years old. Download FIG S1, PDF file, 0.2 MB. Copyright © 2019 Wang et al. 2019 Wang et al. This content is distributed under the terms of the Creative Commons Attribution 4.0 International license.

10.1128/mSystems.00321-18.8 TABLE S1 Basic information on the study subjects. Download Table S1, XLSX file, 0.01 MB. Copyright © 2019 Wang et al. 2019 Wang et al. This content is distributed under the terms of the Creative Commons Attribution 4.0 International license.

### Gut metagenomic composition of ASD.

**FIG 1:**
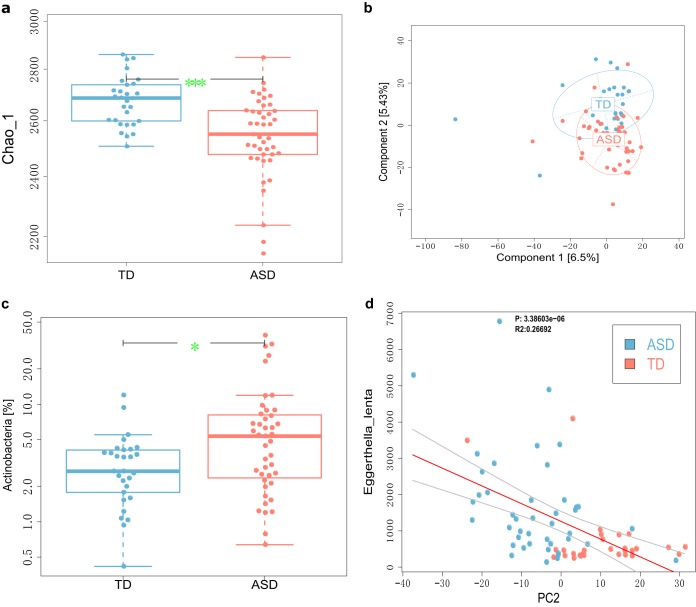
Altered gut microbiota composition in children with ASD. (a) The Chao 1 value for gut microbiota is significantly lower in the ASD group than in the TD group; this means a lower richness in gut microbiota in children with ASD. ***, P < 0.001 by Wilcoxon rank sum test. (b) Principal-component analysis of the gut microbiota composition at the species level shows that the two principal components (PC1 and PC2) can explain 6.5% and 5.43% of the variation, respectively. Distinctions among the groups are clearer at the PC2 level. (c) The abundance of the phylum *Actinobacteria* is significantly higher in children with ASD than in children in the TD group. *, P < 0.05 by Wilcoxon rank sum test. (d) PC2 (bacterial species level) is significantly negatively correlated with Eggerthella lenta abundance.

The gut microbiota alpha diversity was compared between children in the ASD and TD groups. We found a significantly lower species richness in the guts of children with ASD compared with children with TD (*P* value of <0.000111 [Fig. 1a]). To determine whether the gut microbiota can effectively distinguish between the groups, a principal-component analysis (PCA) was performed, with principal component 1 (PC1) and PC2 explaining 6.5% and 5.43% of the variation, respectively. Furthermore, PC2 could effectively distinguish between children with ASD and TD (Fig. 1b). This distinction between ASD and TD in children based on gut microbiota was also confirmed by NMDS analysis (Fig. S2a and b). Next, we explored the difference in gut microbiota at both the taxonomical and functional levels and found significantly higher levels of the phylum *Actinobacteria* in children in the ASD group than in children in the TD group (*P* value of <0.05 [Fig. 1c]). The abundance of Eggerthella lenta in the phylum *Actinobacteria* was significantly negatively correlated with PC2 (Fig. 1d).

10.1128/mSystems.00321-18.2 FIG S2 Gut metagenomic and metabolomic data sets can distinguish between ASD children and TD children. (a and c) NMDS results of the metagenomic and metabolomic data sets, respectively. As can be seen from the figure, the distinguishing effect between the ASD and TD groups is obvious. (b and d) Boxplot of comparison of NMDS2 (b) and NMDS1 (d) of the metagenomic data sets between the ASD and TD groups. ***, *P* value of less than 0.001 by the Wilcoxon rank sum test. Download FIG S2, PDF file, 0.1 MB. Copyright © 2019 Wang et al. 2019 Wang et al. This content is distributed under the terms of the Creative Commons Attribution 4.0 International license.

To understand the differences in the structural and functional composition of the gut microbiota between children in the ASD and TD groups, an MWAS was performed, and 19 significantly different taxons were found; 11 taxons were significantly higher in children in the ASD group compared with those in the TD group, including three *Clostridium* taxons, Clostridium botulinum A3 strain Loch Maree, Clostridium botulinum Ba4 strain 657, and Clostridium cellulolyticum, two *Eggerthella* taxons, Eggerthella lenta and Eggerthella lenta DSM 2243, and two *Klebsiella* taxons, Klebsiella pneumoniae and Klebsiella pneumoniae subsp. *pneumoniae*. Eight taxons were significantly lower in children in the ASD group than in the TD group, including the highly abundant Bacteroides vulgatus*, Betaproteobacteria,* Campylobacter jejuni subsp. *jejuni* 81-176, Campylobacter jejuni subsp. *jejuni* ICDCCJ07001, “*Candidatus* Chloracidobacterium thermophilum B”, Coraliomargarita akajimensis DSM 45221, Proteus mirabilis, and HI4320 Spirochaeta thermophila DSM 6192. Among the 14 significantly different functional pathways, 11 were significantly elevated in children in the ASD group compared with the TD group, including pathways associated with benzoate degradation and tyrosine metabolism pathways. Three pathways were significantly lower in the ASD group, including pathways related to d-glutamine and d-glutamate metabolism and neurotransmitter metabolism. The differential gut microbiota compositions are listed in Table S2.

10.1128/mSystems.00321-18.9 TABLE S2 Gut metabolites and taxons responsible for distinguishing ASD children from TD children. Download Table S2, XLSX file, 0.01 MB. Copyright © 2019 Wang et al. 2019 Wang et al. This content is distributed under the terms of the Creative Commons Attribution 4.0 International license.

To further understand the biological significance of these differences in gut microbiota composition, we performed correlation and regression analyses. As expected, the differences in gut microbiota taxons and pathway were associated, it is worth noting that the significantly higher abundance of the potentially harmful bacteria Clostridium botulinum and Eggerthella lenta and lower abundance of Bacteroides vulgatus in children with ASD were correlated with higher levels of gut microbiota of d-glutamine and d-glutamate metabolism pathways (Fig. S3a), and aromatic amino acid metabolism pathway: benzoate degradation (Fig. S3b), naphthalene degradation (Fig. S3c) and tyrosine metabolism pathways (Fig. S3d).

10.1128/mSystems.00321-18.3 FIG S3 Correlations between taxons and function of gut microbiota in children with ASD. (a) d-Glutamine and d-glutamate metabolism, a neurotransmitter metabolic pathway, are positively associated with Bacteroides vulgatus and Eggerthella lenta DSM 2243 and negatively associated with Clostridium botulinum Ba4 strain 657. (b to d) Gut microbiota functional pathways of three aromatic compounds—benzoate degradation (b), naphthalene degradation (c), and tyrosine metabolism pathways (d)—are negatively associated with Bacteroides vulgatus and positively associated with Eggerthella lenta DSM 2243 and Clostridium botulinum Ba4 strain. The *x* axis represents the value of the correlation coefficient, the *y* axis represents the negative logarithm of the correlated *P* value. The red dots represent the taxons with an absolute value of correlation coefficient greater than 0.3 and FDR-corrected *P < *0.05 (or negative logarithm of the correlated *P* value of >1.30), and the size of the dot represents relative abundance after standardization. Download FIG S3, PDF file, 0.1 MB. Copyright © 2019 Wang et al. 2019 Wang et al. This content is distributed under the terms of the Creative Commons Attribution 4.0 International license.

### Gut metabolomic composition of ASD.

**FIG 2:**
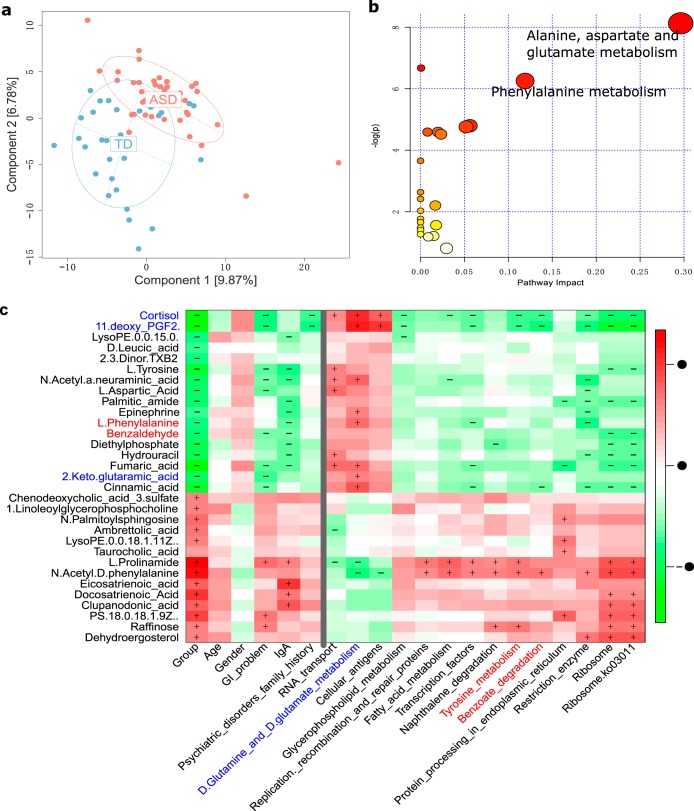
Changes in the gut metabolome of children with ASD are significantly associated with changes in the gut microbiota. (a) PCA analysis of gut metabolome data. The effect of gut metabolites in children with ASD and TD children is effective. (b) Differential gut metabolites can be significantly enriched in alanine, aspartate, and glutamate metabolism and phenylalanine metabolism pathways. (c) Differential metabolites are related to the gut microbiota functional pathway. The gut metabolites l-2-keto-glutaramic acid and aspartic acid are positively associated with the gut microbiota pathways of d-glutamine and d-glutamate metabolism, and gut metabolites benzaldehyde and l-phenylalanine are negatively correlated with gut microbiota pathways of naphthalene degradation and tyrosine metabolism. A color key is shown to the right of the heatmap to show the size of the correlation coefficient; red represents a positive correlation. The + and − symbols in each lattice represent a significant positive and negative correlation, respectively.

To gain a deeper understanding of the composition of the gut microenvironment in children with ASD, a fecal metabolome analysis was performed based on LC-MS/MS. A total of 297 metabolites were detected, among which 123 and 174 were detected in positive mode (ESI+) and negative mode (ESI−), respectively. PCA analysis showed that PC1 and PC2 were able to explain 9.87% and 6.78% of the variation, respectively. Furthermore, children in the ASD and TD groups could be clearly distinguishedon the basis of the metabolites (Fig. 2a and Fig. S2c and S2d).

Through an MWAS performed on the gut metabolome data set, we identified a total of 31 significant differential metabolites between the ASD and TD groups, 14 of which were significantly higher in children in the ASD group, including two bile acid derivatives, taurocholic acid and chenodeoxycholic acid 3-sulfate. The remaining 17 metabolites were significantly lower in the ASD group (Table S2). To interpret the biological meaning of these differences, we performed a pathway enrichment analysis. Interestingly, those metabolites that were significantly lower in children with ASD were associated with alanine, aspartate, and glutamate metabolic pathways (Fig. 2b), including three metabolites: 2-keto-glutaramic acid, l-aspartic acid, and fumaric acid. Thus, the reduction of metabolites in the intestinal microenvironment mainly affects alanine, aspartate, and glutamate metabolism and implies altered neurotransmitter metabolism.

### Gut metagenome-metabolome association.

To understand whether the key changes in the gut metabolites were linked to gut microbiota composition of children with ASD, we first performed a regression analysis of the 31 differential metabolites and metagenome components (PC1/2) and found that around half (16/31) of the metabolites were significantly correlated with PC1/2, and as expected, most (15/16) were significantly correlated with PC2 (data not shown), which may imply that alterations in gut metabolites are linked to changes in gut microbiota composition. Furthermore, we associated the differential metabolites with differential metagenome components; the results showed that not only can most metabolites be correlated with gut microbiota composition at the taxon level (Fig. S4), they can also be correlated with gut microbiota composition at the functional level (Fig. 2c). It is interesting that altered (reduction) gut microbiota glutamate metabolism (Fig. S5 part A) was found and linked to an alteration in the gut hormone 11-deoxy PGF2 (Fig. S5 part B), and changes in the gut hormone cortisol were associated with an increase in potentially harmful bacteria Eggerthella lenta DSM 2243; furthermore, altered aromatic metabolism in the guts of children with ASD were also found. Altered aromatic metabolism was linked to the gut hormone cortisol and associated with changes in the abundance of Bacteroides vulgatus and bacilli (Fig. S5 part C).

10.1128/mSystems.00321-18.4 FIG S4 The difference in gut metabolites and taxons between ASD and TD children are correlated. The *x* axis shows the different taxons found in the ASD and TD children; the *y* axis shows the different metabolic molecules in the ASD and TD children. A color key to the right shows the size of the correlation coefficient. Red represents a positive correlation (the closer to the deep red, the larger the positive correlation coefficient). Green represents a negative correlation (the closer to the dark green, the larger the negative correlation coefficient). The + and − symbols in each lattice represent a significant positive correlation and a significant negative correlation, respectively. Download FIG S4, PDF file, 0.05 MB. Copyright © 2019 Wang et al. 2019 Wang et al. This content is distributed under the terms of the Creative Commons Attribution 4.0 International license.

10.1128/mSystems.00321-18.5 FIG S5 Altered gut metabolites and microbiota. (Part A) Altered glutamate metabolism in the guts of children with ASD. Significant changes (decreased) in gut metabolites were significantly associated with changes in the abundance of gut microbiota glutamate pathway. For each graph, the *x* axis represents the metabolic molecule and the abundance after normalization, the *y* axis represents the gut microbiota pathway and the normalized abundance, and the red and blue dots represent ASD children and TD children, respectively. **(**Part B**)** Altered gut hormone metabolism in children with ASD. (a and b) Changes in the gut hormone cortisol (reduction) are associated with an increase in potentially harmful bacteria Eggerthella lenta DSM 2243 (a) and an decrease in *Betaproteobacteria* (b) abundance. (c) Changes in the gut hormone 11-deoxy PGF2 (reduction) are associated with an decrease in D-glutamine and D-glutamate metabolism microbiota. (d) Changes in the gut hormone cortisol (reduction) are associated with an decrease in tyrosine metabolism microbiota. For each graph, the *x* axis represents the abundance of bacteria and normalization, the *y* axis represents metabolic molecules and standardized abundance, and red and blue dots represent ASD children and TD children, respectively. **(**Part C) Altered aromatic metabolism in gut of children with ASD**. (**a and b**)** Significant changes (increased) in gut microbiota abundance of tyrosine metabolism were associated with changes in the abundance of Bacteroides vulgatus (a) and *Bacilli* (b), respectively. (c) Significant changes (increased) in gut microbiota abundance of benzoate degradation were associated with changes in the abundance of Bacteroides vulgatus. (d) Significant changes (increased) in gut microbiota abundance of naphthalene degradation were associated with changes in the abundance of bacilli. For each graph, the *x* axis represents the gut microbiota pathway abundance after normalization, the *y* axis represents the normalized abundance of gut microbiota taxons, and red and blue dots represent ASD children and TD children, respectively. Download FIG S5, PDF file, 0.2 MB. Copyright © 2019 Wang et al. 2019 Wang et al. This content is distributed under the terms of the Creative Commons Attribution 4.0 International license.

No significant differences in dietary patterns were found between ASD and TD children (Table S3). We evaluated the impact of important clinical phenotypes (e.g., GI problems and gender) on the core differential taxons and metabolites. We found no association between having a GI problem and the presence of Bacteroides vulgatus, Eggerthella lenta, Clostridium botulinum, 2-keto-glutaramic acid, or cortisol (Fig. S6). Similarly, gender had a limited impact on the presence of core differential taxons and metabolites (Fig. S7).

10.1128/mSystems.00321-18.6 FIG S6 Effect of GI problems on significantly different taxons/metabolites in the guts of ASD children and TD children. Part A and Part B show the effect of GI problems on taxons and metabolites, respectively. For each graph, the *x* axis shows “ASD_No”, “ASD_Yes,” and “TD_No”, representing ASD children without GI problems, ASD children with GI problems, and TD children without GI problems, respectively. The *y* axis represents the significantly different gut taxons (Part A) or metabolites (Part B) between the ASD and TD children and normalized abundance. Values that are significantly different by the Wilcoxon rank sum test are indicated by bars and asterisks as follows: *, *P* < 0.05; **, *P* < 0.01; ***, *P* < 0.001. Download FIG S6, PDF file, 0.1 MB. Copyright © 2019 Wang et al. 2019 Wang et al. This content is distributed under the terms of the Creative Commons Attribution 4.0 International license.

10.1128/mSystems.00321-18.7 FIG S7 Effect of gender on significantly different taxons in the guts of ASD children and TD children. Part A and Part B show the effect of gender on taxons and metabolites, respectively. For each graph, the *x* axis shows “ASD_F,” “ASD_M,” “TD_F,” and “TD_M,” representing female ASD children, male ASD children, female TD children, and male TD children, respectively. The *y* axis shows the corresponding gut taxons (Part A) or metabolites (Part B) between ASD and TD and normalized abundance. Values that are significantly different by the Wilcoxon rank sum test are indicated by bars and asterisks as follows: *, *P* < 0.05; **, *P* < 0.01; ***, *P* < 0.001. Download FIG S7, PDF file, 0.1 MB. Copyright © 2019 Wang et al. 2019 Wang et al. This content is distributed under the terms of the Creative Commons Attribution 4.0 International license.

10.1128/mSystems.00321-18.10 TABLE S3 Comparison of diet in ASD and TD children. Download Table S3, XLSX file, 0.01 MB. Copyright © 2019 Wang et al. 2019 Wang et al. This content is distributed under the terms of the Creative Commons Attribution 4.0 International license.

**FIG 3:**
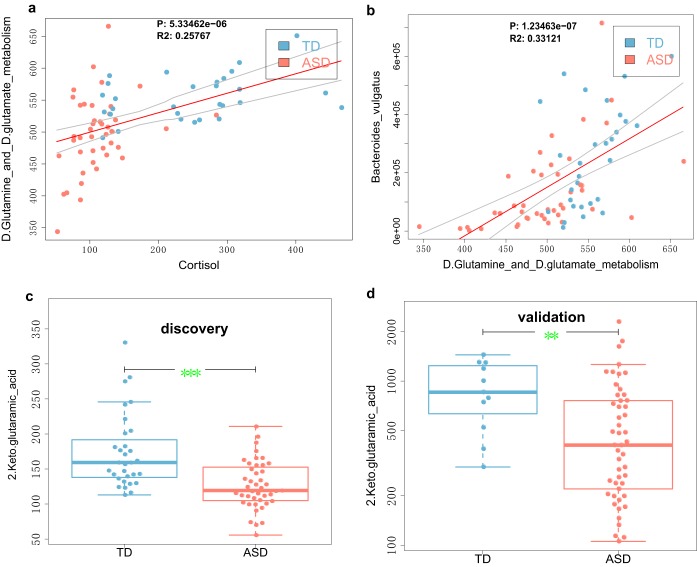
Gut 2-keto-glutaramic acid is a potential marker of ASD. (a) A higher level of gut 2-keto-glutaramic acid is associated with a lower abundance of gut microbiota involved in the d-glutamine and d-glutamate metabolism pathways. (b) A lower abundance of the usually highly abundant Bacteroides vulgatus is significantly correlated with lower levels of d-glutamine and d-glutamate metabolic pathways in the guts of ASD children. (c and d) Significantly lower levels of gut 2-keto-glutaramic acid at the discovery and validation stages, respectively. Values that are significantly different by the Wilcoxon rank sum test are indicated by bars and asterisks as follows: **, P < 0.01; ***, P < 0.001.

Finally, to further validate whether differential gut metabolites are potential biomarkers for ASD, we collected fecal samples from 49 children with ASD and 11 children with TD and tested for differences using LC-MS/MS and a systematic metabolomic analysis. We found that as in the discovery phase, metabolites involved in glutamate metabolism (e.g., 2-keto-glutaramic acid) were significantly lower in the guts of children with ASD (Fig. 3c and d).

## DISCUSSION

Multiple lines of evidence indicate a role for excitatory/inhibitory imbalance, especially abnormalities in the excitatory neurotransmitter glutamate, in the pathogenesis of ASD (7, 8). In support of this, we found that 2-keto-glutaramic acid, l-aspartic acid, and fumaric acid, which are involved in glutamate metabolism, were significantly lower in the guts of children with ASD than in children with TD. Our results are consistent with a study by Tebartz van Elst et al., which found significantly lower glutamate signals in the anterior cingulate cortex and cerebellum of patients with ASD compared with healthy controls (9). Altered gut microbiota associated with glutamate metabolism has been linked to human health. Liu et al. found that a lower abundance of the glutamate metabolic bacteria Bacteroides thetaiotaomicron is linked to obesity (10), and Olson et al. found that the antiseizure effect of the ketogenic diet is linked to gut microbiota-regulated glutamate levels (11). We found that metabolites associated with glutamate metabolism, 2-keto-glutaramic acid, l-aspartic acid, and fumaric acid, are associated with gut microbiota. We also found that a lower abundance of two strains of Campylobacter jejuni, 81-176 and ICDCCJ07001, were associated with lower levels of fumaric acid in the guts of children with ASD. Campylobacter jejuni has been reported to activate glutamate synthesis (12), and therefore, a lower abundance of Campylobacter jejuni may affect the synthesis of glutamate, which in turn would have an indirect impact on glutamate metabolism.

We found that the hypothalamic-pituitary-adrenal (HPA) axis-associated human hormone cortisol was significantly lower in children with ASD than in children with TD. Cortisol is a fear-related hormone synthesized in response to activity in the HPA axis. Abnormal cortisol concentrations have been detected in the sera of patients with ASD (13), and cortisol levels are thus currently considered to be associated with ASD (14, 15); however, little is known about the role of cortisol in the pathogenesis of ASD. We found that lower levels of cortisol in the gut were positively correlated with higher abundance of potentially harmful Clostridium botulinum and Eggerthella lenta and were positively correlated with a lower abundance of Bacteroides vulgatus. Together with previous studies (16), our results suggest that the cortisol-mediated HPA pathway may be involved in the pathogenesis of ASD through altered gut microbiota composition.

We found that aromatic compounds such as epinephrine, benzaldehyde, and cinnamic acid were significantly lower in children with ASD than in children with TD. Epinephrine is a neurotransmitter transformed from tyrosine, and studies have shown that epinephrine can regulate animal behavior, especially fear-related behavior (17), with epinephrine-deficient mice exhibiting impaired conditional fear responses (18). Given that fear and anxiety are important comorbidities for children with ASD (19), we believe that altered epinephrine levels could be associated with ASD. Indeed, in support of this hypothesis, we found that children with ASD have significantly lower epinephrine levels than those with TD. Benzaldehyde is an aromatic compound with antimicrobial and antioxidant functions, and its lower level in the gut may reflect an alteration in gut oxidative stress. We found that the lower levels of benzaldehyde were strongly associated with a higher abundance of the potentially harmful Eggerthella lenta and Clostridium botulinum, which supports the previous hypothesis that children with ASD have defective gut oxidative stress mechanisms and colonization of abnormal microbiota (20). We also found that cinnamic acid, an aromatic compound, was significantly lower in ASD children. Cinnamic acid can be converted from phenylalanine under the catalysis of the enzyme phenylalanine ammonia-lyase; however, phenylalanine ammonia-lyase is a nonmammalian enzyme, found only in plants and bacteria (21, 22).

This study has some innovations. First, the shotgun-based metagenomic sequencing method is conducive to the study of gut microbiota composition at the functional level. Second, conducting metagenome and metabolome studies on the same batch of samples allowed us to identify the gut microbiota composition and metabolites associated with ASD. However, there is limitation in the present study given that it is difficult to distinguish whether the metabolites are from the host or the gut microbiota.

**FIG 4:**
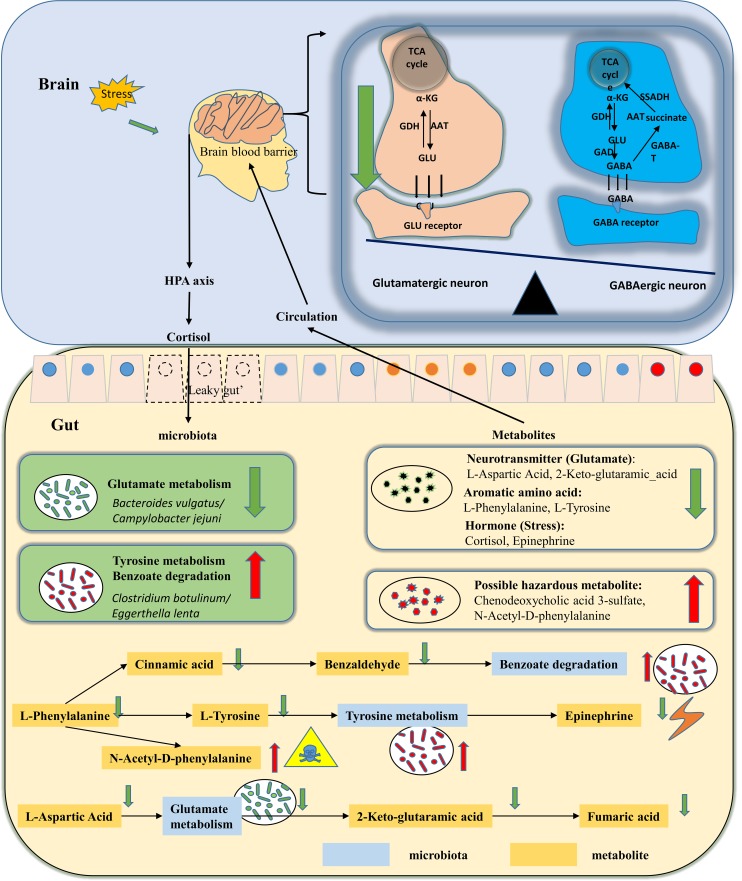
Schematic diagram of the gut pathway and metabolites involved in the pathogenesis of ASD. The gut microenvironment of children with ASD is characterized by altered glutamate metabolism, which is associated with a lower abundance of bacteria such as Bacteroides vulgatus, a species with glutamate metabolism, which may directly affect neurotransmitter inhibition/excitatory imbalance. Children with ASD also exhibit alterations in gut metabolites, such as lower levels of aromatic compounds and higher levels of bile acids, which are related to higher abundance of Eggerthella lenta and Clostridium botulinum. Children with ASD also show altered levels of the stress hormone cortisol, which implies an impact to the gut microbiota via the hypothalamic-pituitary-adrenal (HPA) axis. Abbreviations: AAT, aspartate aminotransferase; α-KG, α-ketoglutaric acid; GABA, gamma-aminobutyric acid; GABA-T, GABA transaminase; GAD, glutamic acid decarboxylase; GLN, glutamine; GLU, glutamic acid; TCA, tricarboxylic acid.

In conclusion, alterations were found in the gut metabolite composition in children with ASD, and these alterations were linked to changes in gut microbiota composition. The hypothesized mechanism based on key gut metabolites and gut microbiota composition (Table 2) are summarized in Fig. 4. In short, we found that children with ASD have abnormalities in aromatic compounds, glutamate metabolism pathways, and bile acid metabolism in the gut, and these changes are associated with a higher abundance of Eggerthella lenta bacteria and Clostridium botulinum and lower levels of Bacteroides vulgatus. These findings are helpful toward the development of new methods for the diagnosis of ASD based on gut microbiota and metabolites.

**TABLE 2:** Key gut microbiota composition and metabolites responsible for distinguishing among children with ASD and children with typical development

Marker names	Marker type	Description	FDR value (Deseq2)	Regulation mode
d-Glutamine and d-glutamate metabolism	Gut microbiota pathways	Glutamate metabolism	5.5893E-05	Down
2-Keto-glutaramic acid	Gut metabolites	Glutamate metabolism	0.009440927	Down
l-Aspartic acid	Gut metabolites	Glutamate metabolism	0.015566567	Down
Bacteroides vulgatus	Gut microbiota taxons	Glutamate-fermenting commensal	0.005836312	Down
Benzoate degradation	Gut microbiota pathways	Aromatic compound metabolism	0.012717845	Up
Naphthalene degradation	Gut microbiota pathways	Aromatic compound metabolism	0.025979256	Up
Tyrosine metabolism	Gut microbiota pathways	Aromatic compound metabolism	0.011855325	Up
Benzaldehyde	Gut metabolites	Aromatic compound metabolism	0.00746784	Down
l-Phenylalanine	Gut metabolites	Aromatic compound metabolism	0.014748155	Down
l-Tyrosine	Gut metabolites	Aromatic compound metabolism	0.004732451	Down
Chenodeoxycholic acid 3-sulfate	Gut metabolites	Gut bile acid metabolism	0.046136738	Up
Taurocholic acid	Gut metabolites	Gut bile acid metabolism	0.028487916	Up
Epinephrine	Gut metabolites	Gut hormone	0.019309289	Down
11-Deoxy PGF2	Gut metabolites	Gut hormone	1.36591E-10	Down
Cortisol	Gut metabolites	Gut hormone	1.99163E-07	Down
Eggerthella lenta	Gut microbiota taxons	Bile acid metabolism taxons	0.013429179	Up
Clostridium botulinum A3 strain Loch Maree	Gut microbiota taxons	Bile acid metabolism taxons	0.021268458	Up
Clostridium botulinum Ba4 strain 657	Gut microbiota taxons	Bile acid metabolism taxons	0.033169563	Up
Eggerthella lenta DSM 2243	Gut microbiota taxons	Bile acid metabolism taxons	0.010687164	Up

## MATERIALS AND METHODS

### Study participants.

A total of 92 children with ASD and 42 children exhibiting TD were recruited at the Shenzhen Children’s Hospital. Forty-three children with ASD and 31 children with TD were enrolled in the discovery stage for both shotgun metagenome sequencing and metabolome analyses, and an additional 49 children with ASD and 11 children with TD were enrolled in the validattion stage for metabolome analyses. The inclusion and exclusion criteria for ASD and TD were as described in our previous study (23) and are detailed in the supplemental material. A diagnosis of ASD was based on guidelines set out in the *Diagnostic and Statistical Manual of Mental Disorders*, 5th edition. The protocol for clinical information collection, including GI problems was detailed in our previous study (24), in which the Rome IV criteria for functional gastrointestinal disorders (25) was used for evaluating GI problems. Dietary habits were investigated through questionnaires. Stool samples were collected from each participant and stored at −80°C before use. The study protocol was in accordance with the Declaration of Helsinki and was approved by the Ethics Committee of Shenzhen Children’s Hospital. Written informed consent was obtained from the children’s parents.

### Shotgun metagenome sequencing and analysis.

Shotgun metagenome sequencing was performed as described in our previous study (26). Briefly, we used the StoolGen fecal DNA extraction kit (CWBiotech Co., Beijing, China) to extract genomic DNA, which was quantified using NanoDrop 2000 (Thermo Scientific, Foster City, CA, USA). A total of 5 µg (or more) of DNA was required for library construction using the TruSeq DNA sample preparation kit (Illumina, San Diego, CA, USA). Libraries were sequenced using an Illumina Hiseq4000 sequencer (Illumina). Metagenome analysis was carried out according to our previous studies (26, 27) and detailed in the supplemental material.

The gut microbiota diversity analysis was performed using Vegan’s diversity function in the R package at species level data. For alpha diversity-based comparison, the Chao 1 value was computed by Vegan’s estimate function. The diversity values for children with ASD and TD are displayed using the boxplot function in R package. For beta diversity-based comparison, principal-component analysis was performed using the ade4 function in R package, and the top two principal components are shown using the dudi.pca function in the R package. Nonmetric multidimensional scaling (NMDS) was performed using Bray-Curtis distance.

To identify gut microbiota composition associated with ASD, a metagenome-wide association analysis (MWAS) was performed according to our previous study (28). The taxonomic composition at the kingdom, phylum, class, order, family, genus, and species levels were used. Briefly, the Wilcoxon rank sum test method and DEseq2 (29), which consider the relative ranking to normalize the basic data were used to identify gut microbiota taxonomic and functional composition associated with ASD. The cutoffs for significant differential taxonomic composition were as follows: mean relative abundance of >0.002%, and false-discovery rate (FDR)-corrected *P* values of ≤0.05 for both Wilcoxon rank sum test and Deseq2. The cutoffs for significant differential pathways were as follows: mean relative abundance of >0.1%; FDR-corrected *P* values of ≤0.05 for both Wilcoxon rank sum test and Deseq2; coverage of >0.99; and |Deseq2 log fold change| of ≥0.15.

### Liquid chromatography-mass spectrometry analysis.

Liquid chromatography-mass spectrometry (LC-MS) was performed according to a previous study (30) and the method is detailed in the supplemental material. In short, metabolites were first separated by liquid chromatography using the Acquity Ultra Performance LC-QTOF system (Waters Corporation, Milford, MA, USA) and further detected using quantitative time-of-flight mass spectrometry (Waters Corporation). The acquired data were processed using Masslynx 4.1 software (Waters Corporation) to obtain a two-dimensional data matrix containing retention time (RT), positive mode or negative mode mass-to-charge ratio (MZ), observations (sample), and peak intensity. Differential gut metabolites between children in the ASD and TD groups were selected according to the Wilcoxon rank sum test and the Deseq2 difference analysis, with cutoffs of FDR-corrected *P* values of ≤0.05, |Deseq2. log fold change| of ≥0.45, and mean metabolite abundance of ≥0.08%. Differential metabolite-associated pathways were enriched using the on-line metaboanalyst 4.0 software (https://metaboanalyst.ca) (31).

### Metagenome-metabolome association analysis.

The linear relationship between metabolites and gut microbiota composition was evaluated using the lm function in R’s regression analysis package and Spearman rank correlation analysis between gut metabolites, taxons abundance, and microbiota pathways were evaluated using the cor and cor.test functions in the R package. FDR-corrected *P* values of less than 0.05 and an absolute value of Spearman correlation coefficient of more than 0.3 were considered significant.

### Data availability.

The metagenome sequencing data set has been uploaded to European Nucleotide Archive under accession no. PRJEB23052. The LC-MS data sets generated and algorithms/scripts used to analyze in this study are available from the corresponding author upon request.
