# Supplementary figures and images for: Alterations in Gut Glutamate Metabolism Associated with Changes in Gut Microbiota Composition in Children with Autism Spectrum Disorder
Source: mSystems. 2019 Jan 29;4(1):e00321-18. doi: 10.1128/mSystems.00321-18 (PMC6351726; doi:10.1128/mSystems.00321-18)

Discovery stage

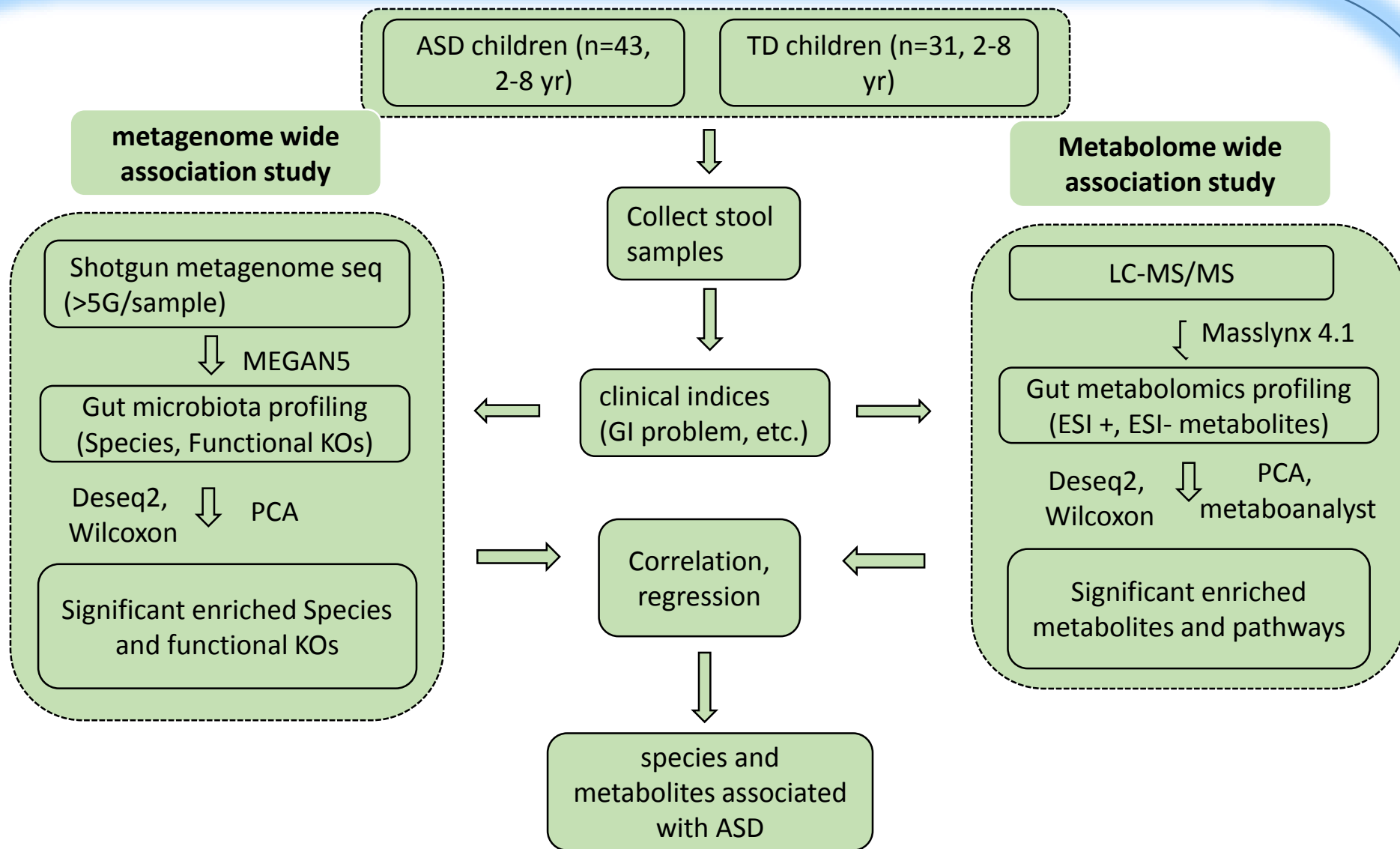

Validation stage

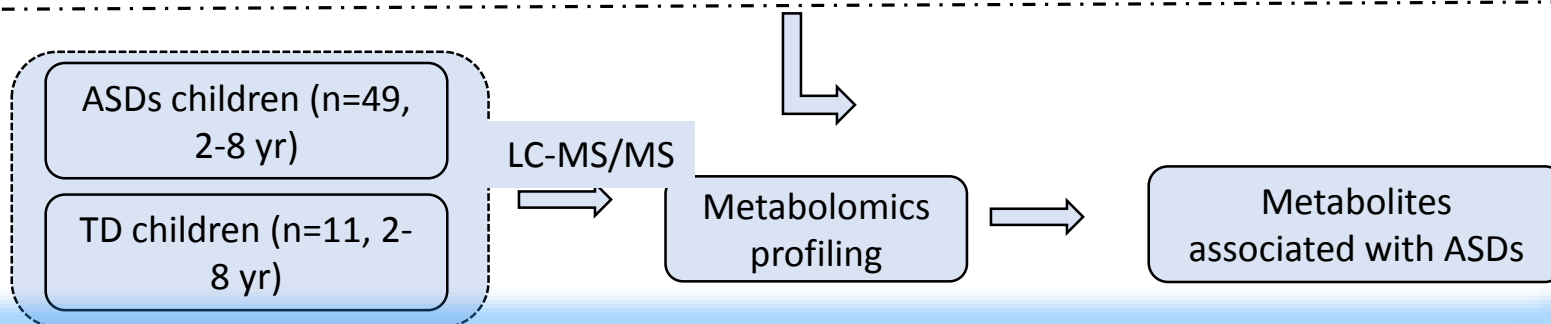

Supplement: FIG S1 [file mSystems.00321-18-sf001.pdf]

**a**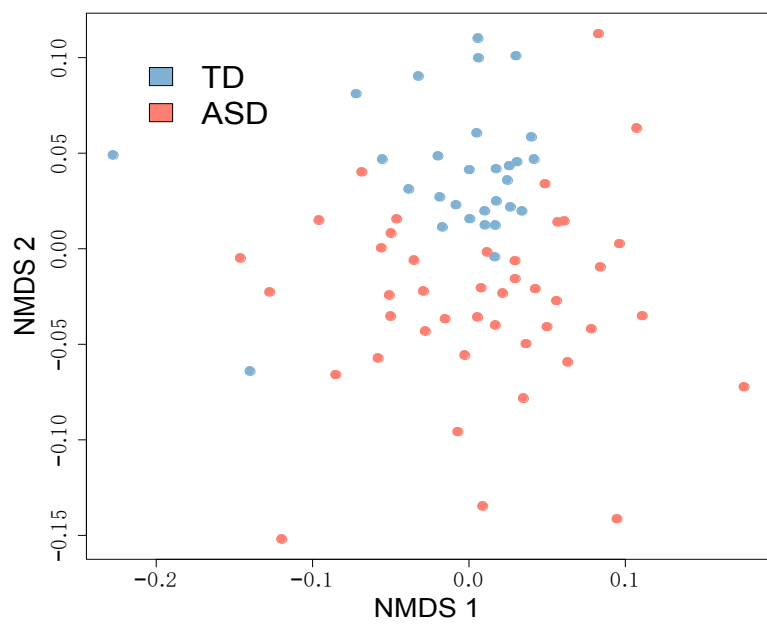**b**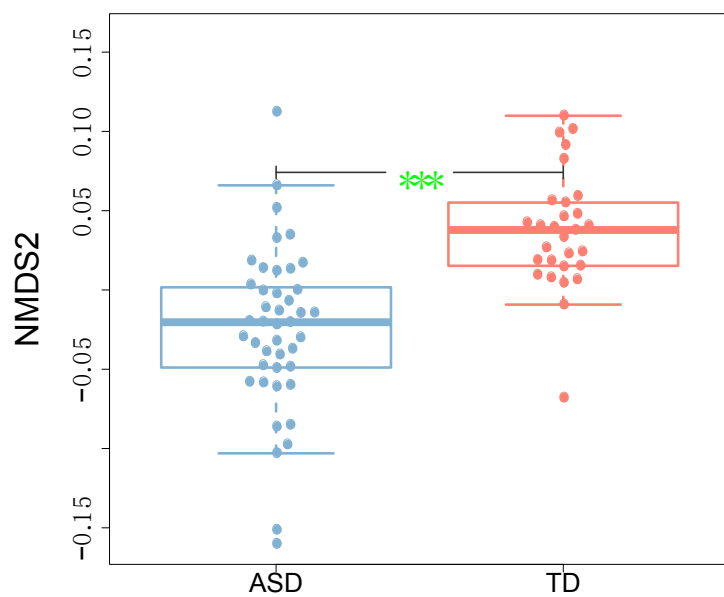**c**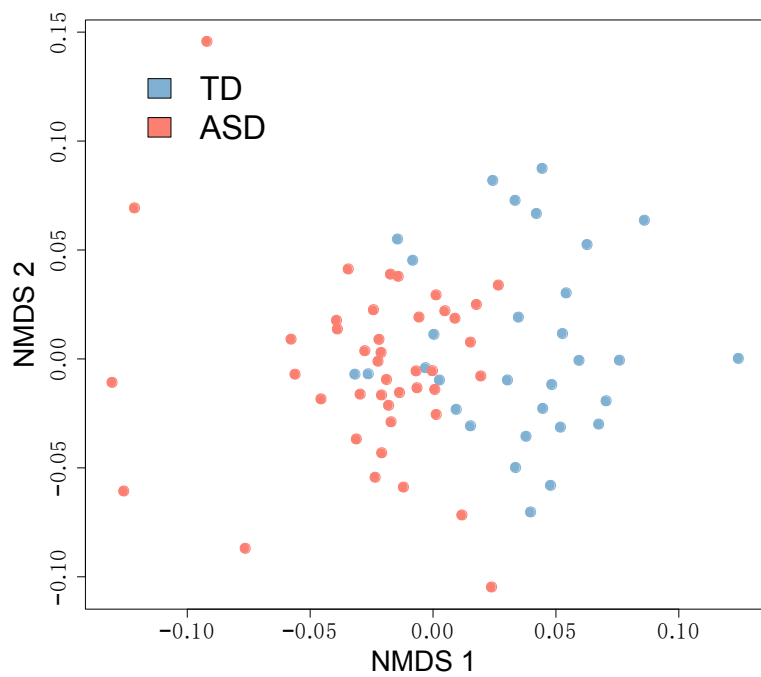**d**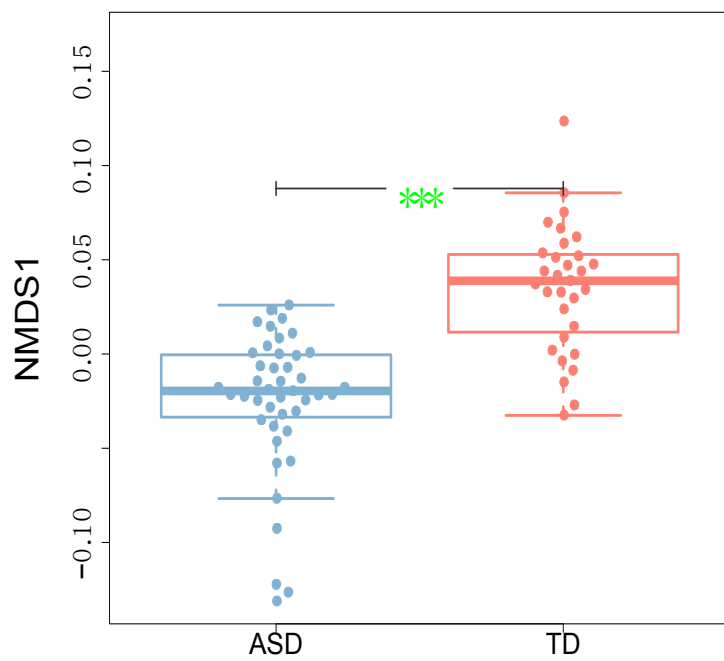

Supplement: FIG S2 [file mSystems.00321-18-sf002.pdf]

a

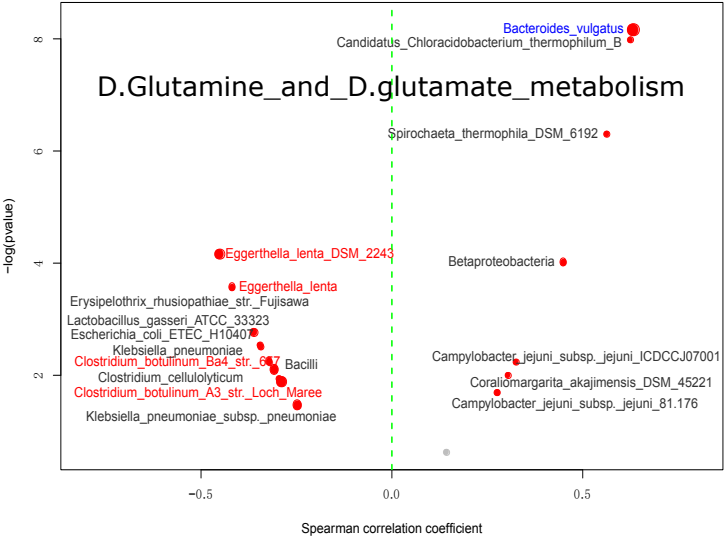

b

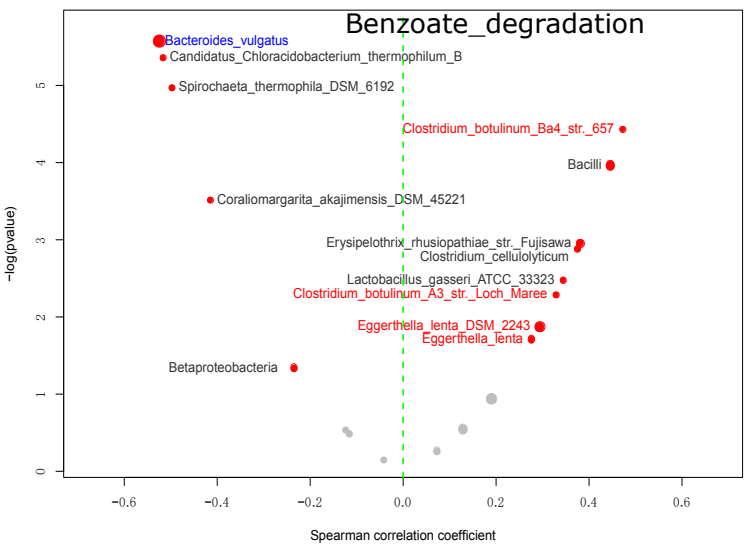

c

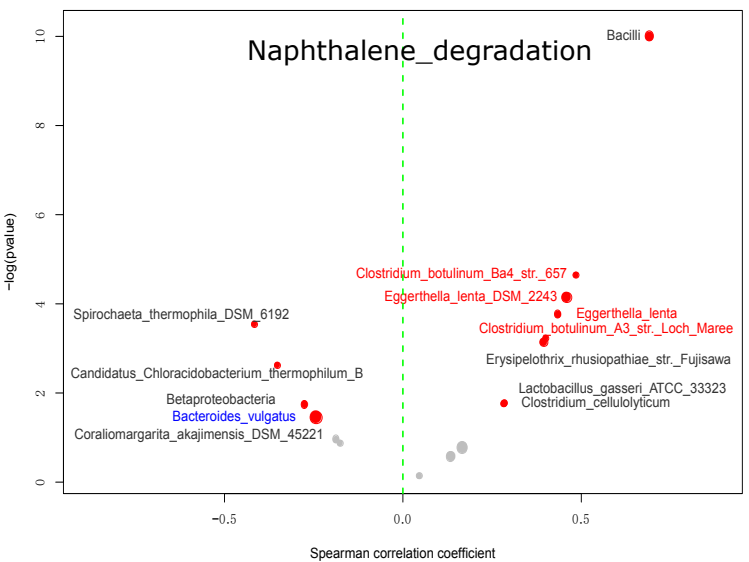

d

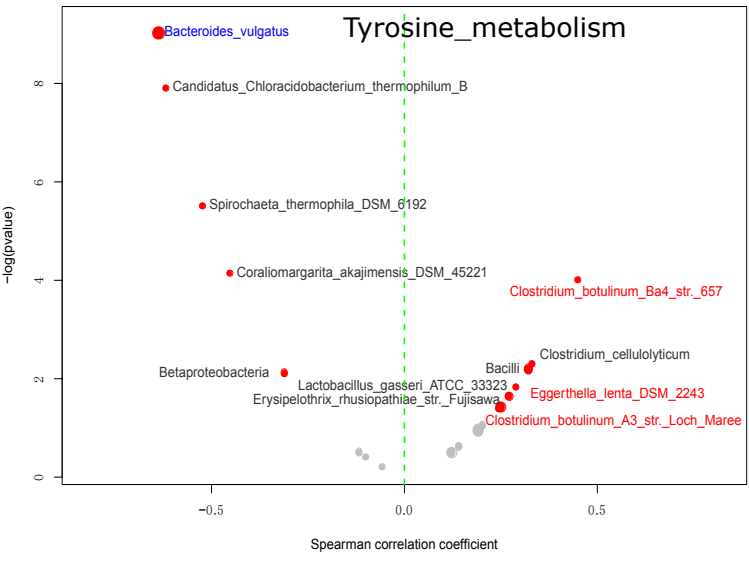

Supplement: FIG S3 [file mSystems.00321-18-sf003.pdf]

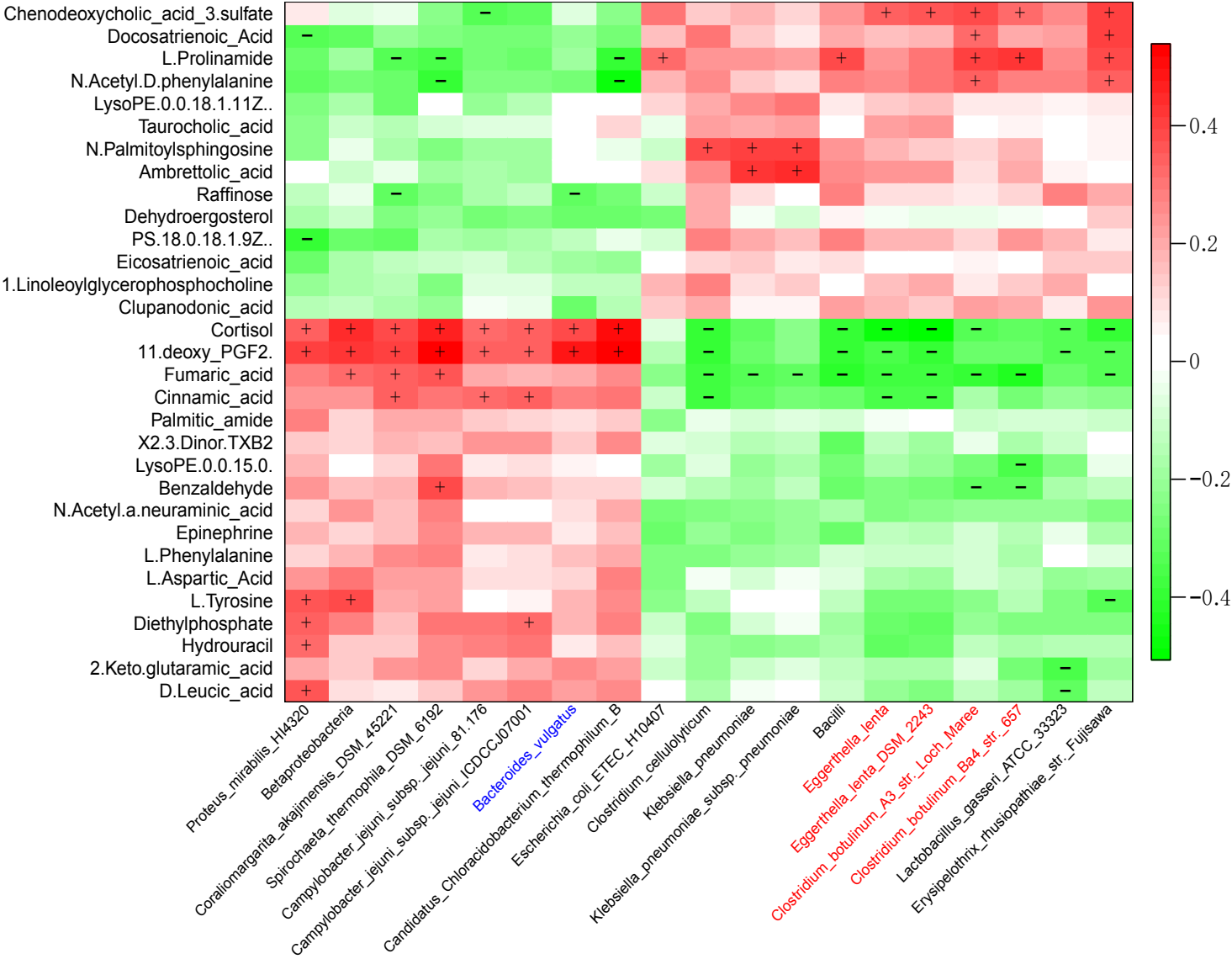

Supplement: FIG S4 [file mSystems.00321-18-sf004.pdf]

## Part A. GI problems effect on microbiota

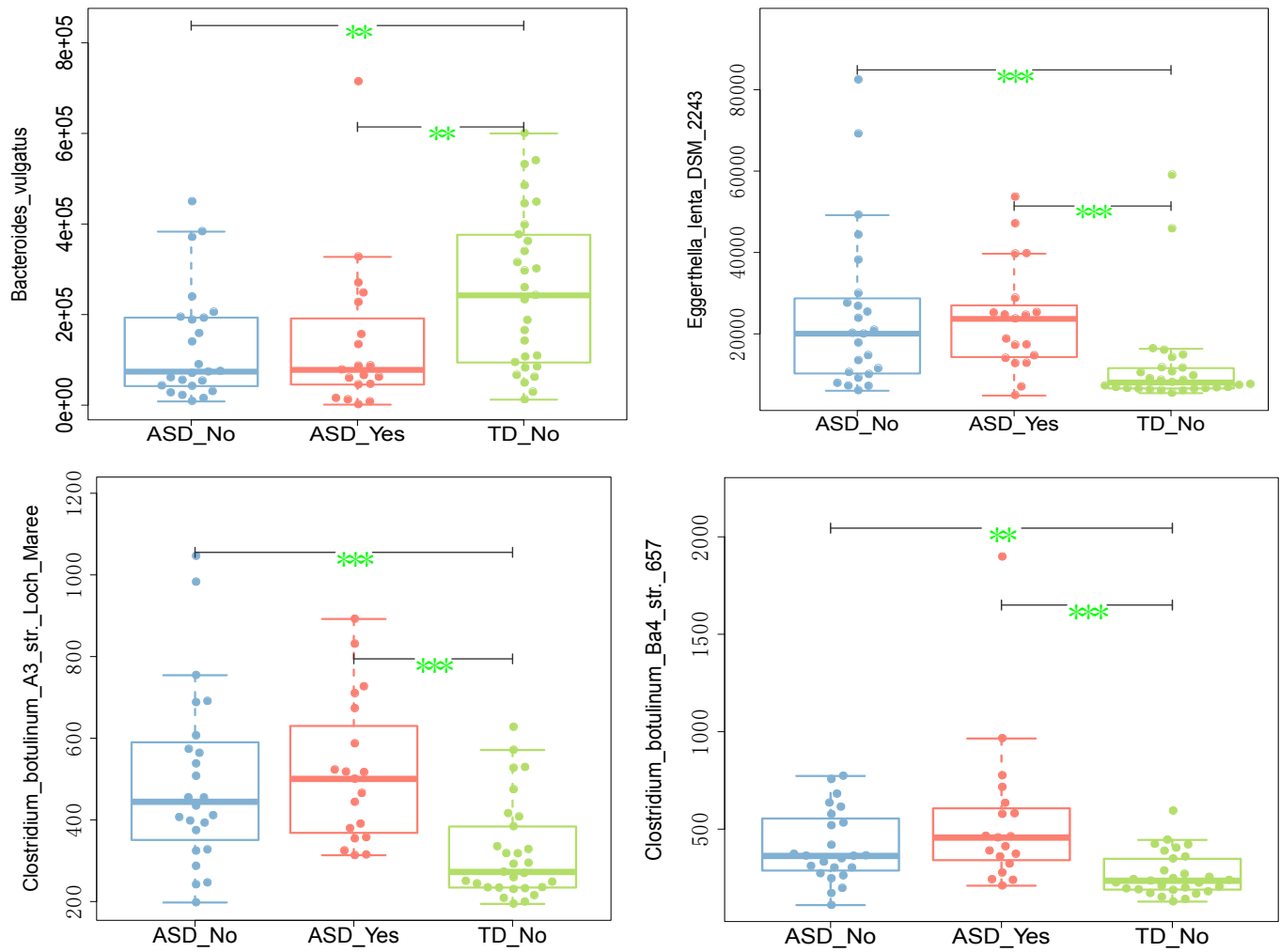

## Part B. GI problems effect on metabolite

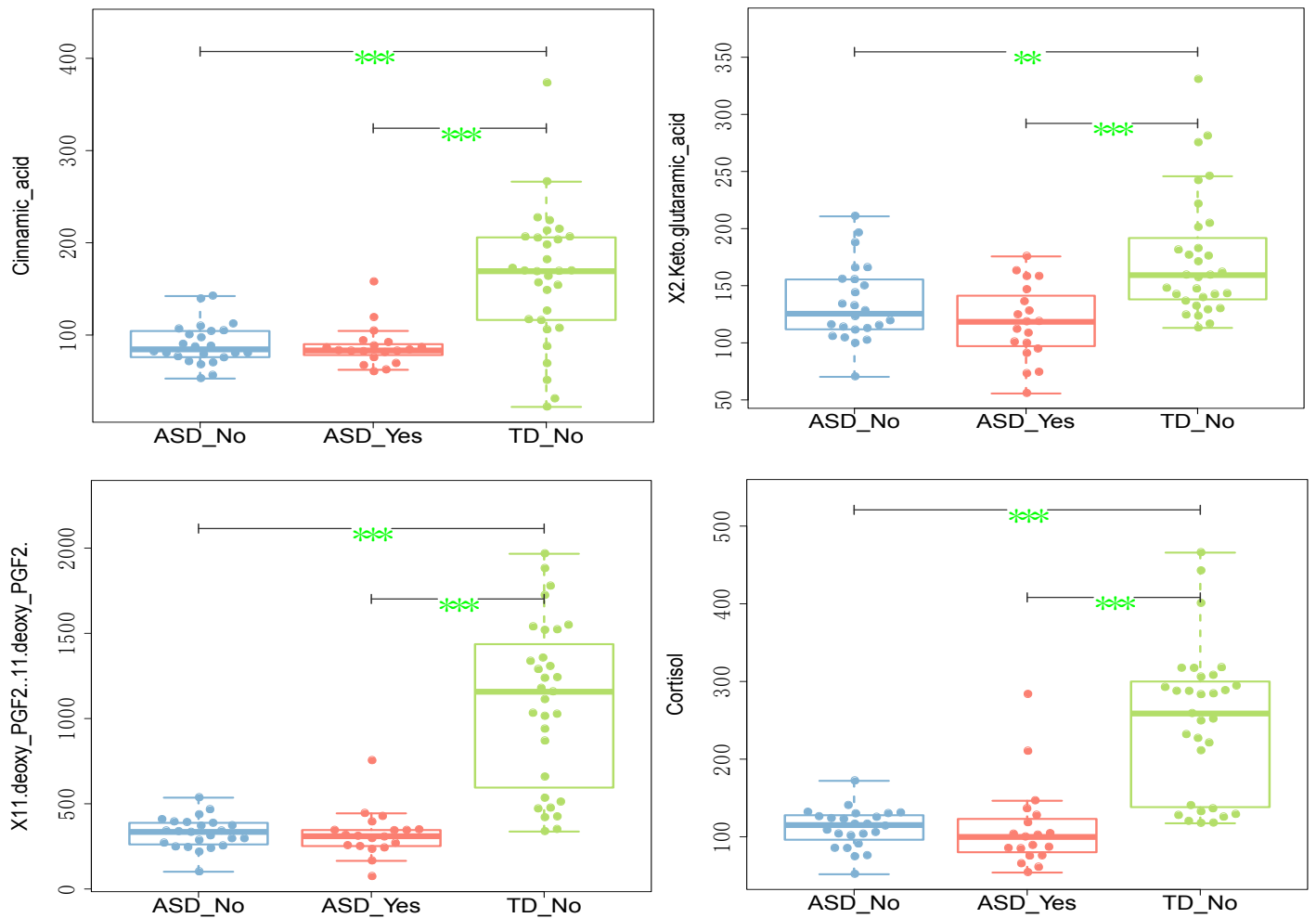

Supplement: FIG S6 [file mSystems.00321-18-sf006.pdf]

## Part A. effect of gender on microbiota

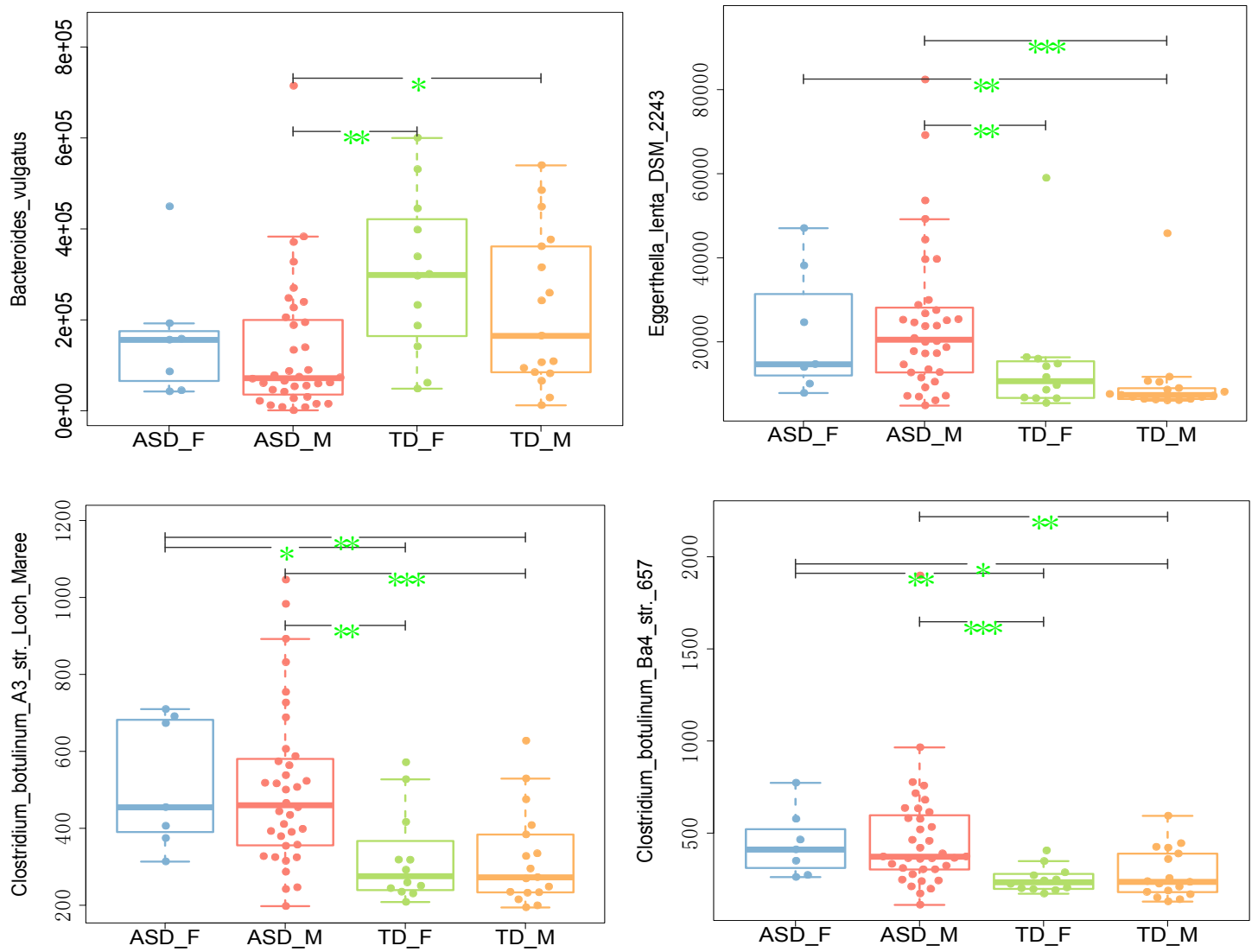

## Part B. effect of gender on metabolites

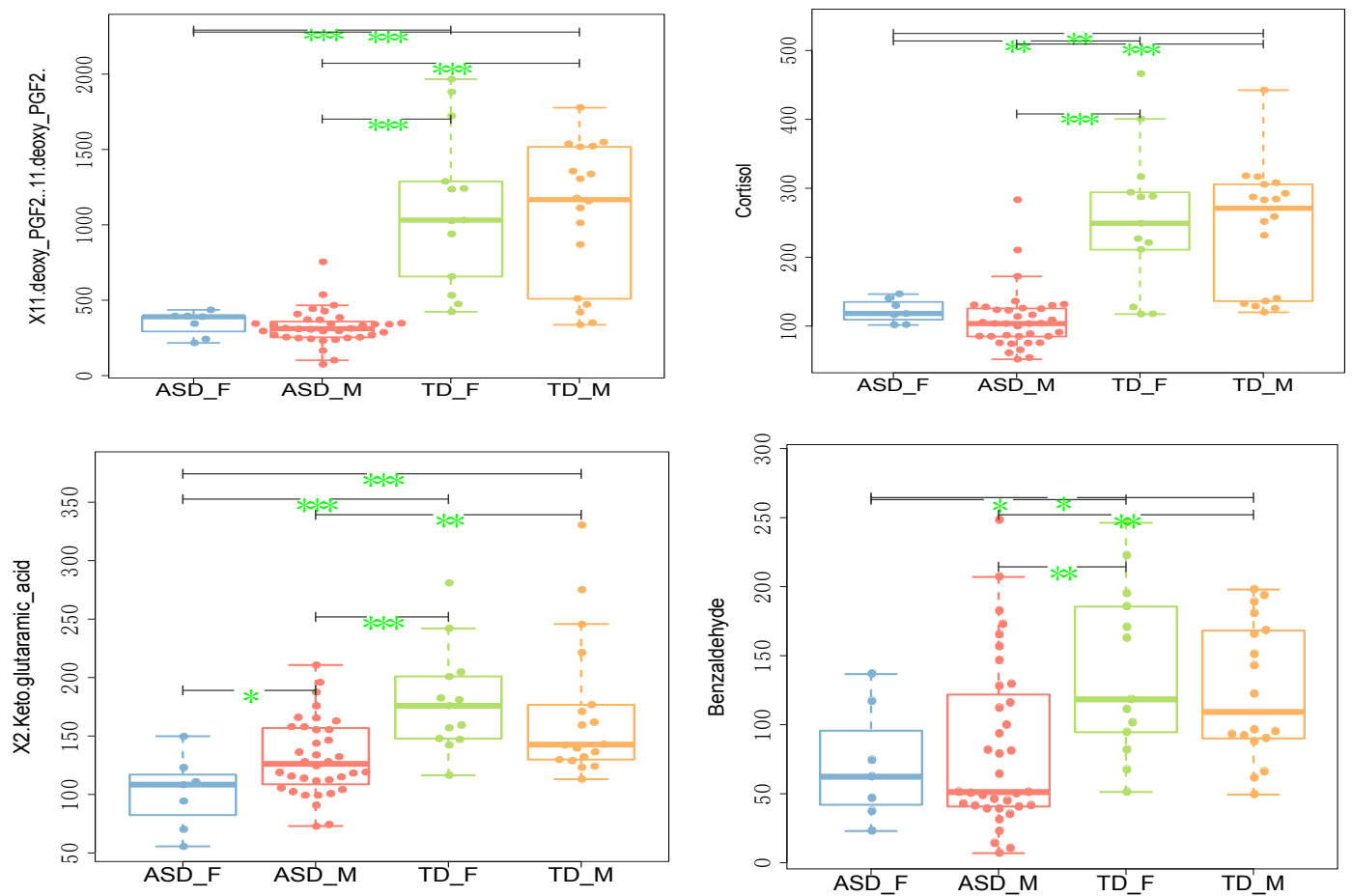

Supplement: FIG S7 [file mSystems.00321-18-sf007.pdf]
