# Supplementary material for: Alterations in Gut Glutamate Metabolism Associated with Changes in Gut Microbiota Composition in Children with Autism Spectrum Disorder
Source: mSystems. 2019 Jan 29;4(1):e00321-18. doi: 10.1128/mSystems.00321-18 (PMC6351726; doi:10.1128/mSystems.00321-18)

Part A. altered gut metabolites and microbiota glutamate metabolism

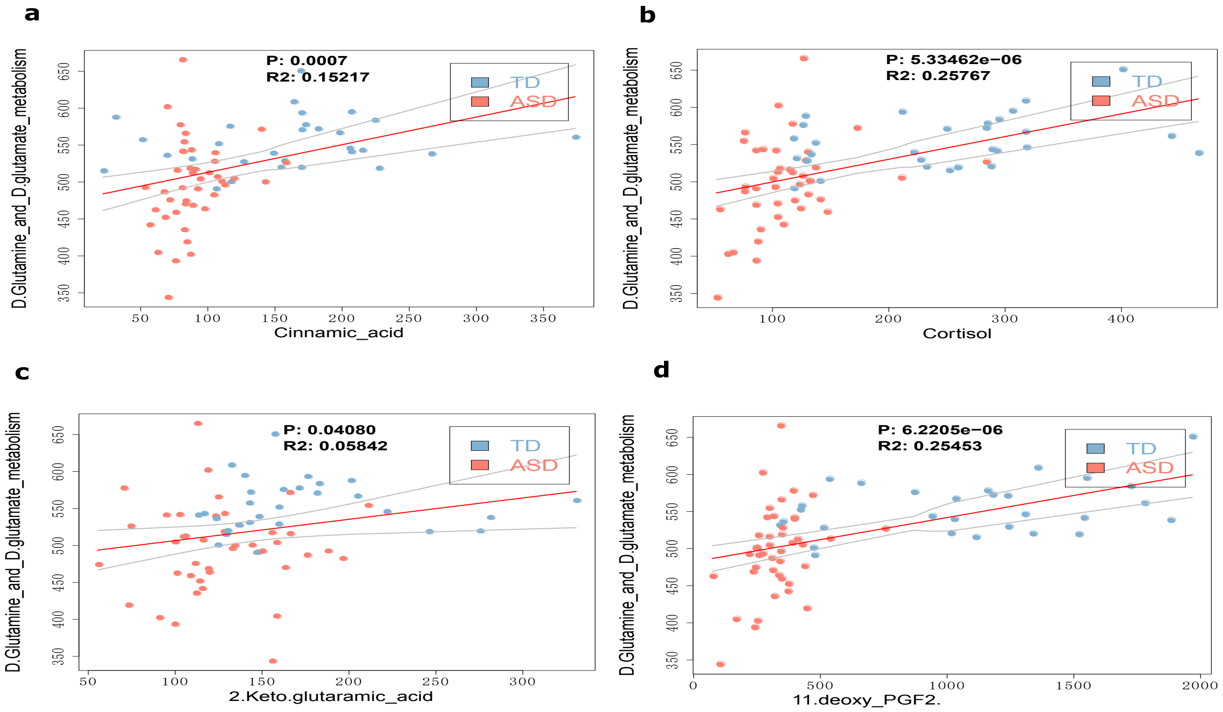

Part B. Gut hormones and microbiota

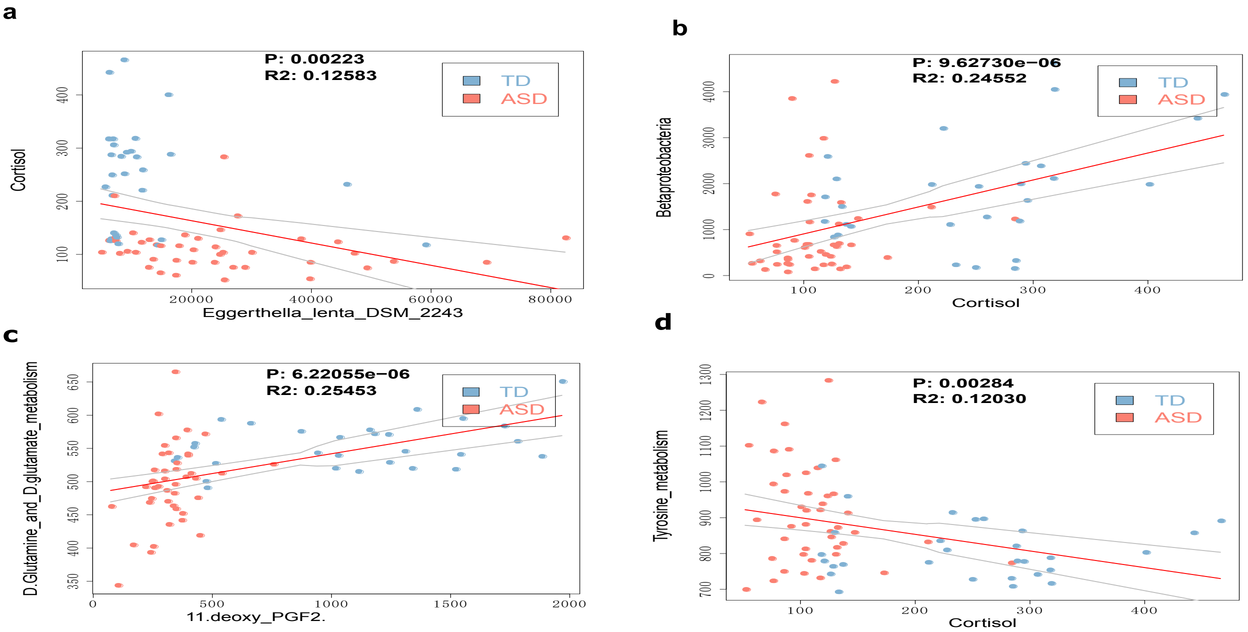

Part C. Gut aromatic metabolites and microbiota pathways

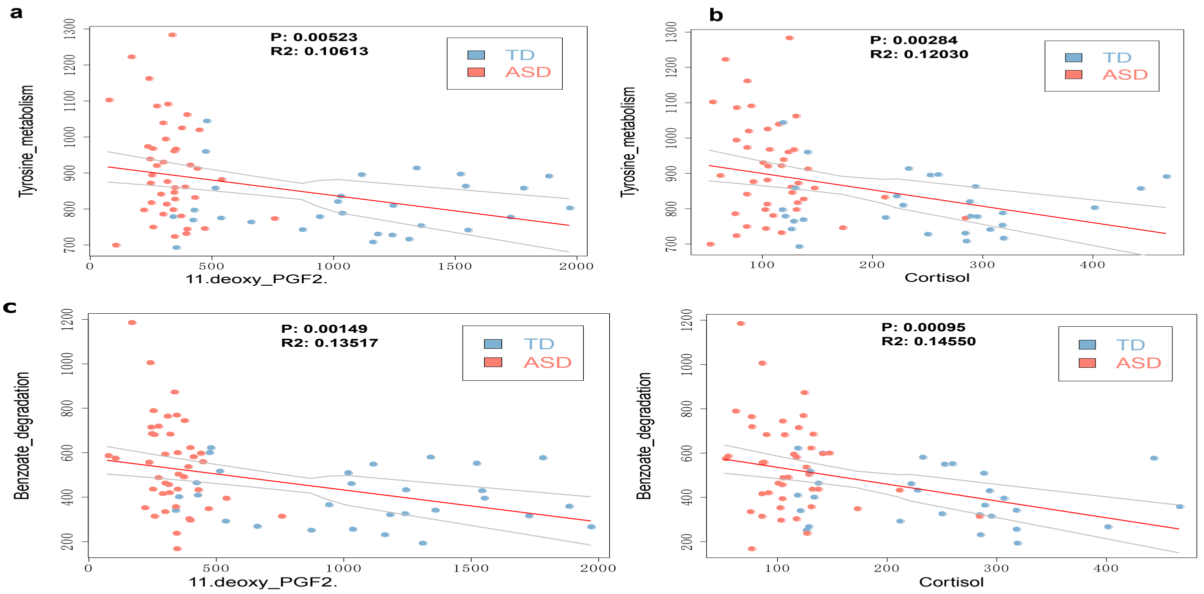

Supplement: FIG S5 [file mSystems.00321-18-sf005.pdf]
